# Association of laparoscopic colectomy versus open colectomy on the long-term health-related quality of life of colon cancer survivors

**DOI:** 10.1007/s00464-019-07360-2

**Published:** 2020-01-28

**Authors:** Melissa S. Y. Thong, Lina Jansen, Jenny Chang-Claude, Michael Hoffmeister, Hermann Brenner, Volker Arndt

**Affiliations:** 1 Unit of Cancer Survivorship, Division of Clinical Epidemiology and Aging Research, German Cancer Research Center (DKFZ), P.O. Box 101949, 69009 Heidelberg, Germany; 2 Division of Clinical Epidemiology and Aging Research, German Cancer Research Center (DKFZ), Heidelberg, Germany; 3 Division of Cancer Epidemiology, German Cancer Research Center (DKFZ), Heidelberg, Germany; 4 Genetic Tumour Epidemiology Group, University Medical Center Hamburg-Eppendorf, University Cancer Center Hamburg, Hamburg, Germany; 5 Division of Preventive Oncology, German Cancer Research Center (DKFZ) and National Center for Tumor Diseases (NCT), Heidelberg, Germany; 6 German Cancer Consortium (DKTK), German Cancer Research Center (DKFZ), Heidelberg, Germany

**Keywords:** Colon cancer, Health-related quality of life, Laparoscopy, Long-term survivor, Population based, Propensity score

## Abstract

**Background:**

Laparoscopic colectomy (LC) is a less invasive alternative to open colectomy (OC) in the treatment of stage I–III colon cancer. Research on the long-term (5-year post-diagnosis) health-related quality of life (HRQOL) of LC patients is scarce. Our study aimed to compare the long-term HRQOL and psychological well-being of stage I–III colon cancer survivors treated either with LC or OC.

**Methods:**

This study used a German population-based cohort of patients treated with either LC (n = 86) or OC (n = 980). LC patients were matched to OC patients using a propensity score. At 5-year follow-up, patients completed assessments on HRQOL (EORTC QLQ-C30 and EORTC QLQ-CR29) and psychological well-being (distress and disease/treatment burden). Least square mean scores of HRQOL were derived using linear regression. Proportions of patients with moderate/high distress and disease/treatment burden were compared with Chi-square tests.

**Results:**

In total, 81 LC patients were matched to 156 OC patients. Generally, LC patients had HRQOL comparable to OC patients, albeit LC patients reported significantly better body image (87.1 versus 81.0, p = 0.03). Distress levels were generally low and comparable between the two groups, even though LC patients were more likely to experience disease recurrence (16% versus 7%, p = 0.02) than OC patients. OC patients were more likely to feel moderate/high levels of burden associated with the treatment (72% versus 56%, p = 0.01) and the time after treatment completion (43% versus 28%, p = 0.02).

**Conclusion:**

LC patients reported comparable long-term HRQOL outcomes but higher levels of psychological well-being than OC patients 5 years after diagnosis, even though LC was associated with higher risk of disease recurrence.

Laparoscopic colectomy (LC) is increasingly adopted as a less invasive alternative to traditional open colectomy (OC) for the curative treatment of stage I–III colon cancer. Results from randomized clinical trials show that LC had more favorable short-term clinical outcomes such as less peri-/post-operative complications and shorter hospital stay, and was comparable with OC in disease-free survival [1–4]. Population-based studies reported better oncologic outcomes favoring LC, in both the short and long term [5–8].

Regarding health-related quality of life (HRQOL), results are mixed. LC has been associated with better peri-operative, post-operative, and short-term HRQOL when compared with OC [9–12]. Also, HRQOL tended to improve back to or surpass pre-LC levels within a year [13]. Conversely, some studies have reported no significant differences in HRQOL between LC and OC patients [14–16]. However, these studies were randomized clinical trials, based on a single institution, had small samples or reported on short-term HRQOL (< 5-year post-surgery). The only randomized clinical trial with up to 5 years follow-up, published so far, reported comparable HRQOL between LC and OC [17]. To our knowledge, there are no published population-based results on the long-term HRQOL (5-year post-surgery) of colon cancer survivors treated either with LC or OC.

The use of LC for the treatment of colon cancer is encouraged in view of its better clinical outcomes and cost-effectiveness when compared with OC [18, 19]. However, a recent study on colorectal cancer patients showed that perceptions of surgical outcomes differed between patients and surgeons; patients reported that being cured of colorectal cancer and avoiding complications were more important compared to factors traditionally considered by surgeons such as use of laparoscopy, incision appearance or length of hospital stay [20]. LC could be associated with higher disease recurrence [21] although results from randomized controlled trials and population-based studies have indicated otherwise [17, 19]. However, we found no published reports on the fear of recurrence of patients treated with LC. Research with breast cancer patients suggest that fear of recurrence is an important motivator for choosing mastectomy over breast conservation therapy [22]. As such, it is important to have a clearer picture of the consequences of LC on long-term HRQOL and psychological well-being of patients.

Therefore, the aims of the current study are twofold: to compare the (1) long-term HRQOL and (2) psychological well-being of stage I–III colon cancer survivors by treatment (LC versus OC).

## Methods

### Setting and participants

We used data from the population-based case–control DACHS (Darmkrebs: Chancen der Verhütung durch *S*creening) study. The DACHS study was started in 2003 in collaboration with 22 hospitals located in the Rhine-Neckar region of southwest Germany, an area with a population of approximately 2 million. To date, DACHS has included over 6000 cases (‘patients’) and continues to recruit individuals with a newly diagnosed and histologically confirmed primary colorectal cancer (ICD 10: C18-20). Other inclusion criteria include being at least 30 years of age, German speaking, and being physically and mentally able to participate in a baseline interview of approximately one hour. Further details of the DACHS study have been reported elsewhere [23].

The DACHS study was approved by the ethics committees of the University of Heidelberg and the state medical boards of Baden-Württemberg and Rhineland-Palatinate. Written informed consent was obtained from all participants.

### Data collection

Eligible patients were identified by their treating clinician during their hospital stay or were contacted by mail shortly after discharge by clinicians or clinical cancer registries. Information was collected at baseline, and at 3- and 5-year follow-ups. At baseline, trained interviewers used a standardized questionnaire to collect detailed socio-demographic, clinical, and lifestyle history. Detailed treatment and recurrence information were provided by the attending physicians at the 3-year follow-up. At 5-year follow-up, 86% of patients still alive completed a mailed questionnaire on HRQOL and changes in medical or recurrence history (Supplementary Fig. 1). Attending physicians verified self-reported recurrence or new cancers (‘disease recurrence’) at 5-year follow-up. Vital status of participants was checked via population registries after 3- and 5-year post-diagnosis. The current study reports on patients diagnosed with stage I-III colon cancer treated with either LC or OC between 2003 and 2014 and have completed a HRQOL questionnaire at 5-year follow-up between 2009 and 2016.

#### HRQOL

HRQOL was assessed with the European Organization for Research and Treatment of Cancer Quality of Life Core-30 (EORTC QLQ-C30) questionnaire [24] and the colorectal cancer-specific module (EORTC QLQ-CR29) [25, 26]. The EORTC QLQ-C30 consists of five functioning scales (physical, role, cognitive, emotional, social), a global health/quality of life (QOL) scale, and nine items/scales on symptom and financial impact. The EORTC QLQ-C29 consists of functioning (anxiety, body image, weight concerns, sexual interest—male, sexual interest—female) and symptom scales (urinary frequency, urinary incontinence, dysuria, blood and mucus in stool, stool frequency, abdominal pain, buttock pain, bloating, dry mouth, hair loss, taste, flatulence, fecal incontinence, sore skin, embarrassment, stoma care problems, impotence, dyspareunia). The EORTC QLQ-CR29 subscales sexual interest-male and sexual interest-female were combined into the subscale ‘sexual interest,’ as were the subscales impotence (male) and dyspareunia (female) which were combined into the subscale ‘sexual problems.’ Answers ranged from 1 (not at all) to 4 (very much), and from 1 (very poor) to 7 (excellent) for items in the global health/QOL scale. All raw scores were linearly transformed to scales of 0–100 using standard procedures [27]. Higher functioning and global health/QOL scores indicated better function or health status; higher scores on symptom items/scales and financial impact indicated more symptom complaints and greater financial impact.

#### Psychological well-being

##### Questionnaire on distress in cancer survivors (QSC-R10)

The 10-item QSC-R10 is a validated instrument assessing distress experienced by cancer survivors in daily life. For this study, we used three items which are relevant for our sample, namely feeling physically imperfect, fear of disease progression, and not being able to participate in hobbies as before cancer [28]. Items score ranged from 0 (‘not applicable’) to 5 (‘a very serious problem’) [28].

#### Burden due to cancer and treatment

Four items assessed patients’ perceptions of burden due to cancer and treatment. Items included the burden of initial diagnosis, with treatment, with time after completion of treatment, and with follow-up consultation and investigations. Items score ranged from 1 (‘not at all’) to 4 (‘very’).

### Statistical analyses

#### Propensity score matching

To address possible confounding common in observational studies, a propensity score was calculated to indicate the probability of being treated with LC [29]. Logistic regression was used to derive the propensity score conditional to a set of baseline covariates which included age at diagnosis, gender, tumor stage, tumor location (proximal or distal), hospital volume, presence of comorbid condition (heart failure, angina pectoris, hypertension, diabetes mellitus), education, employment status, place of residence (village, town, city), and lifestyle factors (body mass index (BMI), smoking status, and alcohol consumption) [30–32]. The LC sample was matched on propensity score to the OC sample using a 1:2 nearest-neighbor algorithm with a caliper distance of 0.2 standard deviation [33–35].

Differences in demographic and clinical characteristics between the two groups stratified by surgery were determined with t-test for continuous variables or Chi-square test for categorical variables. Least square means of the EORTC QLQ-C30 and the EORTC QLQ-CR29 scale scores between the samples were derived using linear regression. For single items on psychological well-being, we defined a priori that items on the QSC-R10 with a score of 4 or 5 and items on burden due to cancer/treatment with a score of 3 or 4 indicated moderate-to-high impact on psychological well-being. The difference in proportion of LC and OC patients indicating moderate-to-high psychological impact was tested with Chi-square tests.

#### Sensitivity analyses

To address the reduction in the OC sample size from propensity score matching, we compared the mean EORTC QLQ-C30 and QLQ-CR29 scores of LC and OC patients using the whole sample (unmatched), with the propensity score included as a covariate for adjustment [36]. Other covariates included for adjustment were comorbidity and lifestyle factors (BMI, smoking status, alcohol use) at 5-year follow-up. As disease recurrence could influence HRQOL, we reran the analyses excluding patients with disease recurrence, using the matched and unmatched samples.

All analyses were conducted with SAS (version 9.4 for Windows, SAS Institute Inc., Cary, NC). Statistical significance was determined at *p* < 0.05 (two-sided). The *p* values were not adjusted for multiple testing and referred to the individual tests rather than a global test for differences.

## Results

### Patients’ characteristics

This study included 994 stage I-III colon cancer survivors who completed the HRQOL questionnaire at 5-year follow-up (Supplementary Fig. 1), of whom 86 (9%) were surgically treated with LC. Patients treated with LC were significantly younger at diagnosis than those treated with OC (65.0 ± 10.5 versus 67.8 ± 9.8, *p* = 0.01) (Table 1). LC patients were also more likely to have stage I disease, have a tumor located in the distal colon, treated in a large volume hospital, less likely to have hypertension, were better educated, employed, living in the city, and weight within normal BMI range when compared with OC patients.

**Table 1:** Demographic and clinical characteristics of sample stratified by surgery, before and after propensity score matching

*N* (%)	Unmatched sample			Matched sample		
	Laparoscopy (*n* = 86)	Open colectomy (*n* = 908)	*p* value	Laparoscopy (*n* = 81)	Open colectomy (*n* = 156)	*p* value
Mean age at diagnosis	65.0 ± 10.5	67.8 ± 9.8	0.01	64.9 ± 10.6	65.0 ± 11.3	0.96
Gender			0.88			0.74
Female	37 (43)	398(44)		35 (43)	64 (41)	
Male	49 (57)	510 (56)		46 (57)	92 (59)	
Tumor stage			0.0002			0.76
I	39 (45)	229 (25)		36 (44)	63 (40)	
II	24 (28)	397 (44)		23 (28)	44 (28)	
III	23 (27)	28 (31)		22 (27)	49 (31)	
Tumor location			< 0.0001			0.48
Distal	71 (83)	407 (45)		66 (81)	121 (78)	
Proximal	15 (17)	500 (55)		15 (19)	35 (22)	
Missing	–	1 (0.1)		–	–	
Chemotherapy			0.09			0.07
Yes	21 (24)	302 (33)		20 (25)	56 (36)	
No	65 (76)	606 (67)		61 (75)	100 (64)	
Radiotherapy			0.76			–
Yes	–	3 (0.3)		0	0	
No	86 (100)	905 (100)		81 (100)	156 (100)	
Disease recurrence			0.13			0.02
Yes	13 (15)	87 (10)		13 (16)	11 (7)	
No	73 (85)	820 (90)		68 (84)	145 (93)	
Missing	–	1 (0.1)		–	–	
Hospital volume			< 0.0001			0.37
Small	1 (1)	223 (25)		1 (1)	0	
Medium	38 (44)	297 (33)		36 (44)	72 (46)	
Large	47 (55)	387 (43)		44 (54)	84 (54)	
Missing	–	1 (0.1)		–	–	
Comorbidity at baseline						
Angina pectoris	6 (7)	93 (10)	0.33	5 (6)	11 (7)	0.79
Heart failure	4 (5)	104 (11)	0.05	3 (4)	7 (4)	0.77
Hypertension	35 (41)	489 (54)	0.01	33 (41)	62 (40)	0.88
Diabetes mellitus	10 (12)	148 (16)	0.25	9 (11)	16 (10)	0.83
Marital status at baseline			0.10			0.87
Single	8 (9)	45 (5)		7 (9)	13 (8)	
Married	60 (70)	650 (72)		57 (70)	108 (70)	
Divorced	8 (14)	51 (6)		8 (10)	12 (8)	
Widowed	10 (12)	159 (18)		9 (11)	22 (14)	
Missing	–	3 (0.3)		–	1 (1)	
Education			0.0006			0.98
≤ 9 years	40 (47)	609 (67)		37 (46)	73 (47)	
10–11 years	22 (26)	154 (17)		22 (27)	42 (27)	
> 12 years	24 (28)	145 (16)		22 (27)	41 (26)	
Employment status at baseline			0.0004			0.50
Full-/part-/self-employed	33 (38)	177 (19)		31 (38)	57 (37)	
Housewife/man	10 (12)	93 (10)		9 (11)	10 (6)	
Unemployed/ (early) retired	43 (50)	631 (69)		41 (51)	88 (56)	
Other	–	7 (1)		–	1 (1)	
Place of residence			0.02			0.99
Village (pop: < 10,000)	21 (24)	324 (36)		20 (25)	38 (24)	
Town	27 (31)	307 (34)		26 (32)	51 (33)	
City (pop: > 100,000)	38 (44)	277 (31)		35 (43)	67 (43)	
Body mass index (BMI; kg/m^2^) at baseline			0.04			0.96
< 25 Normal*	39 (45)	312 (34)		36 (44)	67 (43)	
25–30 Overweight	36 (42)	393 (43)		34 (42)	66 (42)	
> 30 Obese	11 (13)	202 (22)		11 (14)	23 (15)	
Missing	–	1 (0.1)		–	–	
Smoking status at baseline			0.55			0.73
Never	35 (41)	422 (46)		32 (40)	64 (41)	
Former	37 (43)	374 (41)		37 (46)	64 (41)	
Current	13 (15)	111 (12)		12 (15)	28 (18)	
Missing	1 (1)	1 (0.1)		–	–	
Alcohol use (g/day) at baseline			0.80			0.96
< 0.9	24 (28)	252 (28)		23 (28)	47 (30)	
0.9–6.1	19 (22)	191 (21)		18 (22)	32 (21)	
6.2–14.4	12 (14)	145 (16)		12 (15)	27 (17)	
14.55–30.7	19 (22)	160 (18)		18 (22)	30 (19)	
> 30.7	11 (13)	146 (16)		10 (12)	20 (13)	
Missing	1 (1)	14 (2)		–	–	

Propensity score derived from baseline covariates including age at diagnosis, gender, tumor stage, tumor location, hospital volume, education, comorbidity, employment status, place of residence, BMI, smoking status, alcohol use

*BMI-normal category includes underweight patients (BMI < 18.5). Both samples had comparable proportions; unmatched sample: LC = 2 (2%), OC = 21 (2%); matched sample: LC = 1 (1%), OC = 5 (3%)

Using the derived propensity score, 81 LC patients were matched with 156 OC patients. No suitable matches were found for 5 LC patients and 6 LC patients had 1 match with OC patients. After matching, both groups were comparable on baseline characteristics (Table 1). However, LC patients were more likely to experience disease recurrence (16% versus 7%, *p* = 0.02) than OC patients.

#### HRQOL

**Fig. 1:**
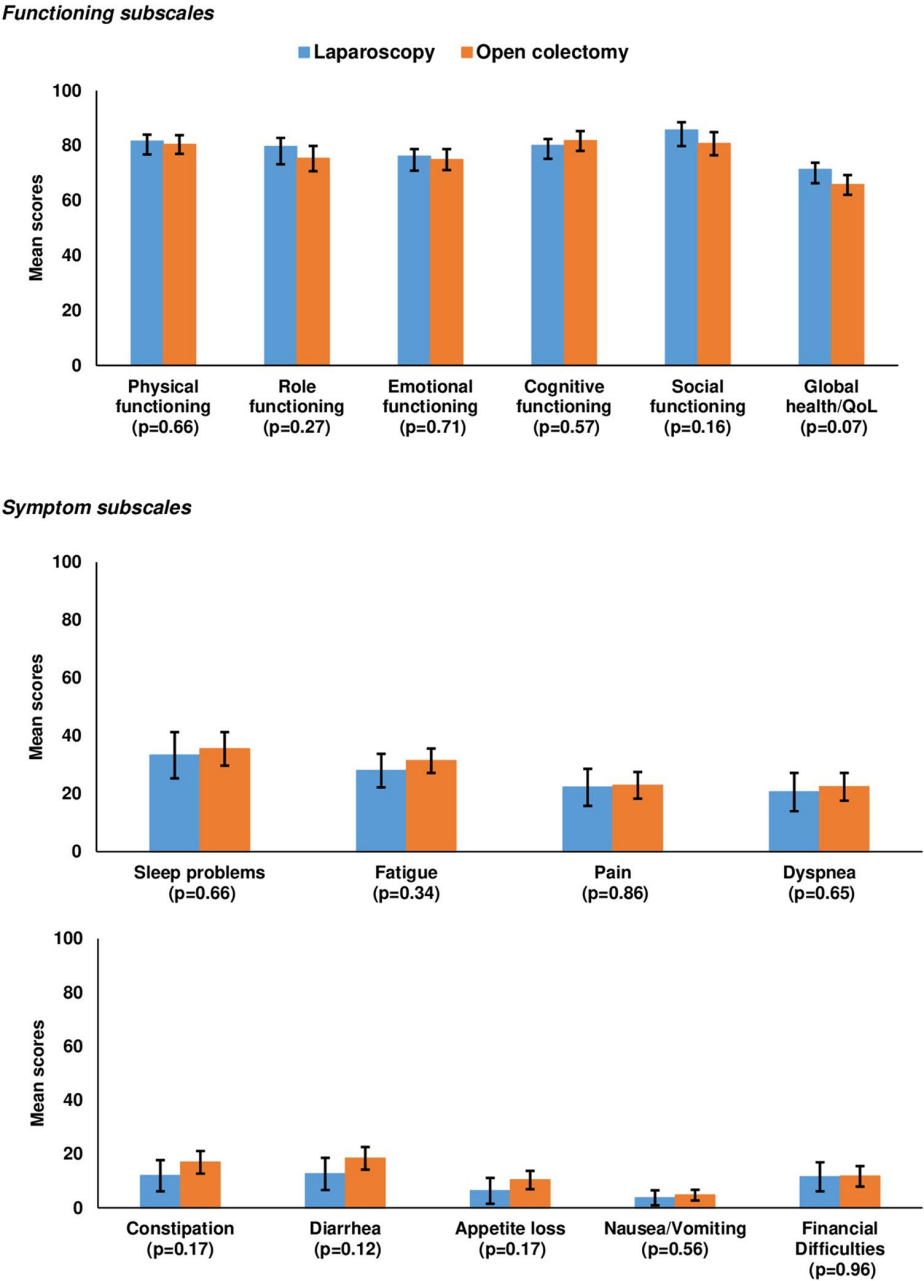
Mean EORTC QLQ-C30 scores of stage I–III colon cancer patients, stratified by treatment and matched by propensity score (LC: *n* = 81, OC: *n* = 156). For functioning subscales, higher scores indicate better functioning; for symptom subscales, higher scores indicate higher symptom burden. Propensity score derived from baseline covariates including age at diagnosis, gender, tumor stage, tumor location, hospital volume, education, comorbidity, employment status, place of residence, BMI, smoking status, alcohol use

**Fig. 2:**
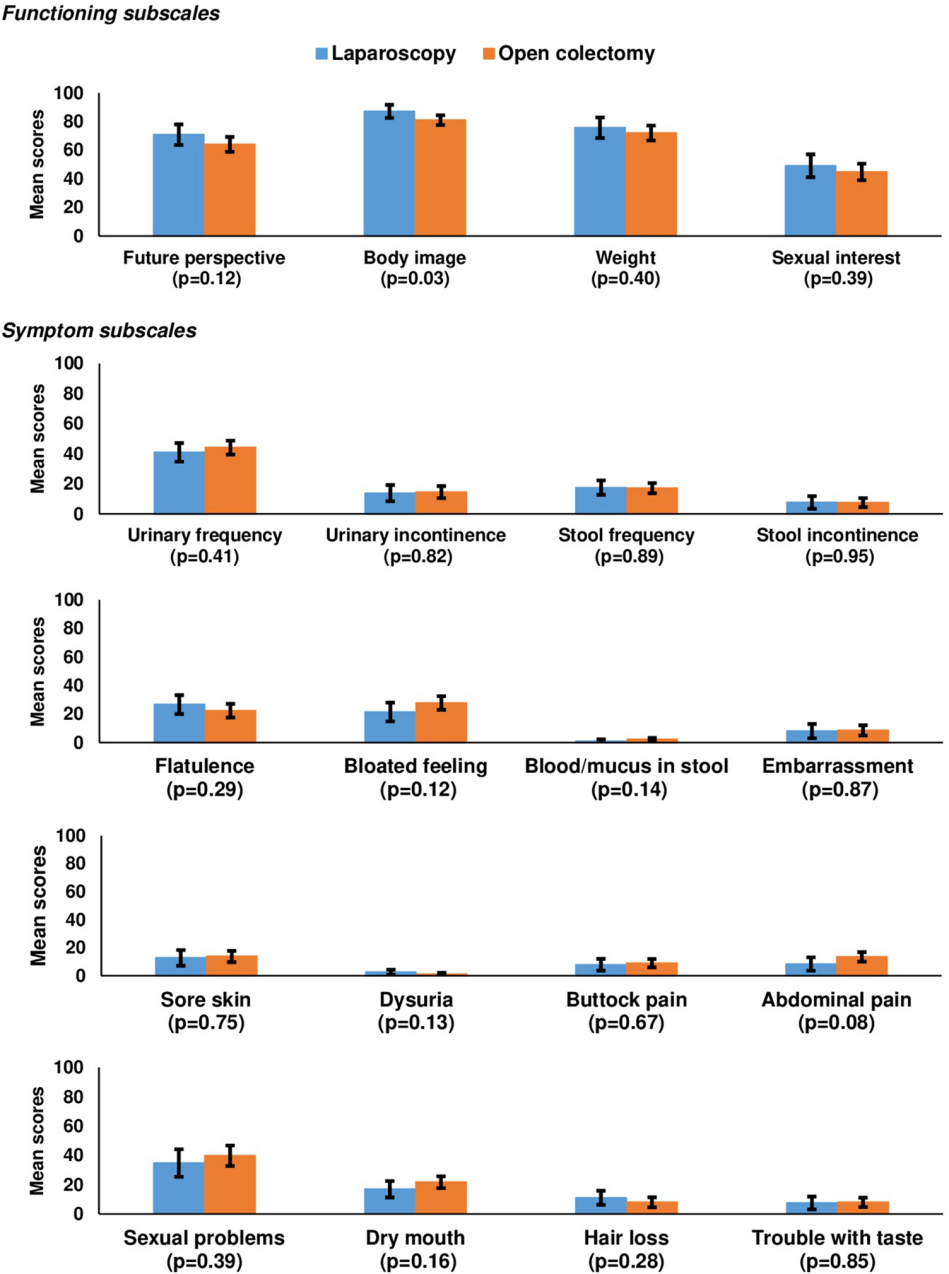
Mean EORTC QLQ-CR29 scores of stage I–III colon cancer patients, stratified by treatment and matched by propensity score (LC: *n* = 81, OC: *n* = 156). For functioning subscales, higher scores indicate better functioning; for symptom subscales, higher scores indicate higher symptom burden. Propensity score derived from baseline covariates including age at diagnosis, gender, tumor stage, tumor location, hospital volume, education, comorbidity, employment status, place of residence, BMI, smoking status, alcohol use

In general, no statistical differences were found for any of the functioning subscales of the EORTC QLQ-C30 and the EORTC QLQ-CR29 except for a higher body image in LC patients (87.1 versus 81.0, *p* = 0.03) (Figs. 1 and 2).

In terms of symptom burden, no statistically significant differences were found (Figs. 1 and 2).

#### Psychological well-being

**Fig. 3:**
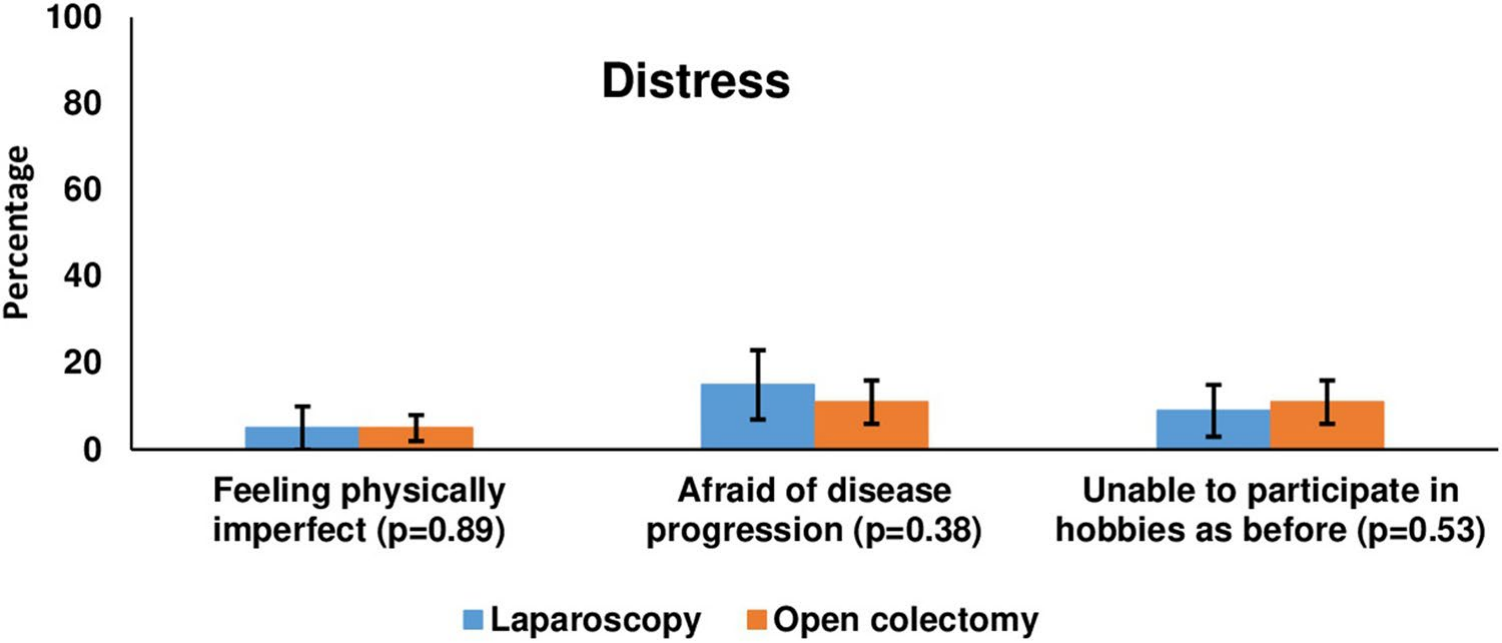
Percentage of patients who scored ≥ 4 on relevant single items of the Questionnaire on Distress in Cancer Survivors (QSC-R10) [28], using propensity score matched sample (LC: *n* = 81, OC: *n* = 156)

**Fig. 4:**
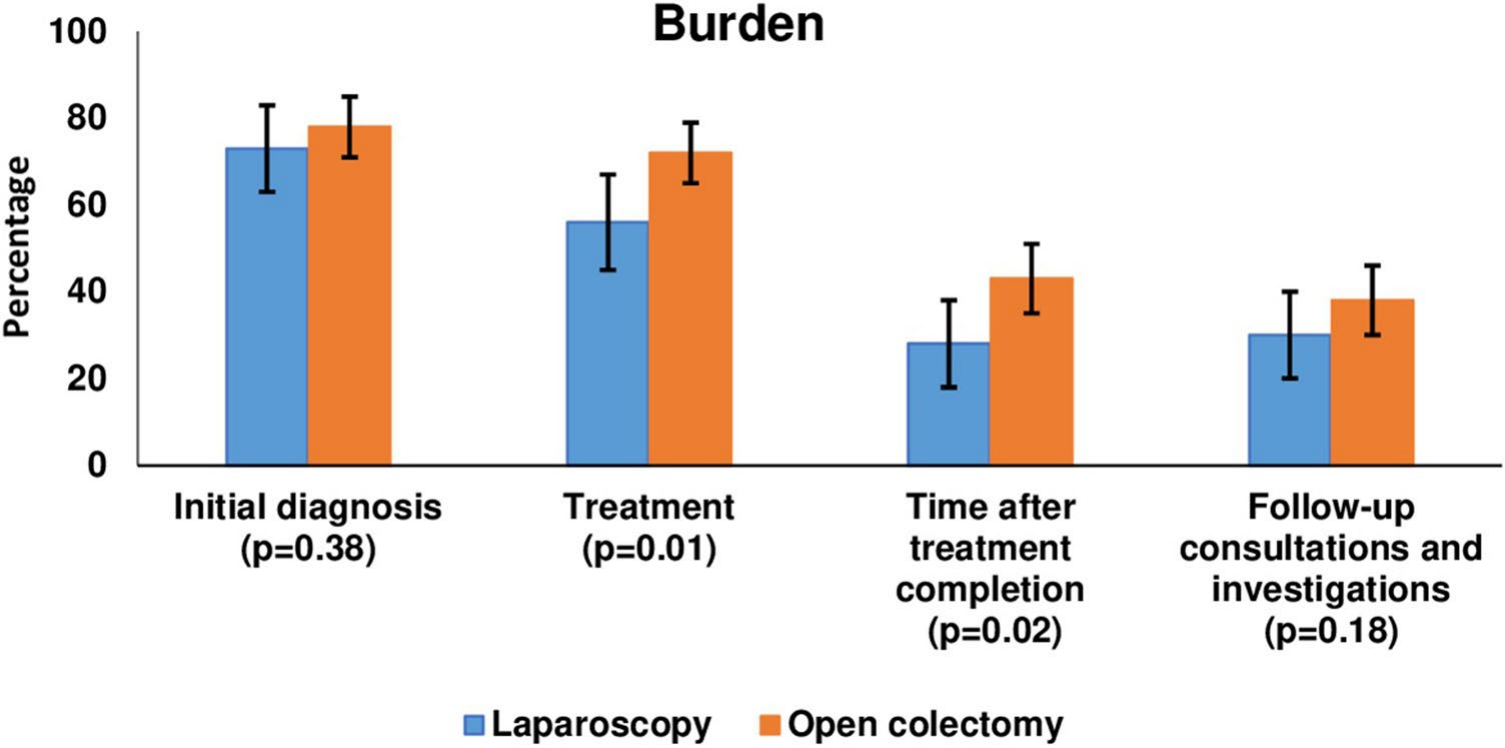
Percentage of patients who scored ≥ 3 on aspects of burden of cancer and its treatment, using propensity score matched sample (LC: *n* = 81, OC: *n* = 156)

No differences of statistical significance were found for the distress items (Fig. 3). With regards to perception of burden of cancer and treatment, OC patients were more likely to feel moderate-to-high levels of burden associated with the treatment (72% versus 56%, *p* = 0.01) and the time after completion of treatment (43% versus 28%, *p* = 0.02) (Fig. 4).

### Sensitivity analysis

Results based on conventional multiple regression using the unmatched sample were generally comparable to that derived from the matched sample. LC patients reported significantly better body image than OC patients (Table 2). Analyses using the matched and unmatched samples but excluding patients with disease recurrence showed results similar to those analyses in which these patients were included (Supplementary Tables 1 and 2).

**Table 2:** Mean HRQOL scores of stage I-III colon cancer patients, stratified by treatment (unmatched sample)

	Laparoscopy (*n* = 83)*				Open colectomy (*n* = 885)*				*p* value
	Mean	SE	95% CL	95% CU	Mean	SE	95% CL	95% CU	
EORTC QLQ-C30									
Functioning									
Physical functioning	77.9	2.9	72.3	83.5	76.1	1.1	73.9	78.3	0.54
Role functioning	77.2	3.8	69.8	84.7	72.9	1.5	69.9	75.8	0.25
Emotional functioning	74.9	3.1	68.9	81.0	72.6	1.2	70.2	75.0	0.45
Cognitive functioning	79.7	3.0	73.9	85.5	78.6	1.2	76.3	80.8	0.71
Social functioning	83.2	3.6	76.1	90.2	77.5	1.4	74.8	80.3	0.12
Global health/QOL	69.3	2.9	63.6	74.9	65.1	1.1	62.9	67.3	0.15
Symptom									
Sleep problems	31.9	4.4	23.2	40.5	35.4	3.0	29.5	41.3	0.50
Fatigue	27.1	3.1	21.1	33.1	30.3	2.1	26.2	34.5	0.38
Pain	22.8	3.3	16.2	29.4	22.4	2.3	17.9	26.9	0.92
Dyspnea	20.6	3.6	13.5	27.6	21.8	2.4	17.0	26.7	0.77
Constipation	13.2	3.3	6.7	19.7	17.5	2.3	13.0	21.9	0.29
Diarrhea	11.3	3.2	4.9	17.6	18.4	2.2	14.1	22.7	0.06
Appetite loss	6.0	2.5	1.0	11.0	9.7	1.7	6.3	13.1	0.23
Nausea and vomiting	3.7	1.4	1.0	6.4	4.3	0.9	2.4	6.1	0.72
Financial difficulties	10.3	2.9	4.6	16.0	11.2	2.0	7.3	15.1	0.79
EORTC QLQ-CR29									
Functioning									
Future perspective	70.6	4.1	62.5	78.6	66.2	1.6	63.1	69.4	0.29
Body image	87.2	2.8	81.7	92.7	81.5	1.1	79.4	83.7	0.04
Weight concerns	72.2	3.8	64.7	79.7	71.3	1.5	68.4	74.3	0.83
Sexual interest	46.6	4.3	38.1	55.2	42.7	1.7	39.4	46.0	0.36
Symptom									
Urinary frequency	43.1	3.4	36.3	49.8	44.6	1.4	42.0	47.3	0.65
Urinary incontinence	15.4	3.3	9.0	21.8	15.7	1.3	13.2	18.2	0.91
Stool frequency	19.2	2.8	13.6	24.7	19.7	1.1	17.5	21.9	0.86
Stool incontinence	8.4	2.8	2.9	13.8	10.2	1.1	8.1	12.4	0.50
Flatulence	24.7	3.9	17.0	32.3	24.9	1.5	21.9	27.9	0.94
Bloated feeling	17.6	3.7	10.2	24.9	24.5	1.5	21.7	27.4	0.06
Blood/mucus in stool	1.5	1.1	-0.8	3.7	3.3	0.5	2.5	4.2	0.10
Embarrassment	9.8	3.1	3.8	15.8	11.0	1.2	8.7	13.4	0.69
Sore skin	14.9	3.0	9.0	20.8	14.9	1.2	12.6	17.2	0.99
Dysuria	4.5	1.6	1.3	7.7	4.3	0.6	3.0	5.5	0.90
Buttock pain	9.5	2.6	4.3	14.7	11.4	1.0	9.3	13.4	0.47
Abdominal pain	7.5	2.8	1.9	13.0	13.1	1.1	10.9	15.2	0.05
Sexual problems	36.7	5.3	26.2	47.2	41.0	2.1	36.9	45.1	0.42
Dry mouth	19.6	3.6	12.6	26.7	26.0	1.4	23.2	28.7	0.08
Hair loss	12.4	3.1	6.4	18.4	10.7	1.2	8.3	13.1	0.58
Trouble with taste	9.1	2.9	3.4	14.9	9.8	1.2	7.5	12.0	0.82

For functioning subscales, higher scores indicate better functioning; for symptom subscales, higher scores indicate higher symptom burden

*Sample excludes those patients for whom a propensity score could not be calculated

95% CL, CU: 95% lower confidence level, upper confidence level

Mean scores are adjusted for propensity score and 5-year follow-up covariates (comorbidity, BMI, smoking status, alcohol use)

Propensity score derived from baseline covariates including age at diagnosis, gender, tumor stage, tumor location, hospital volume, education, comorbidity, employment status, place of residence, BMI, smoking status, alcohol use

## Discussion

This population-based study showed that HRQOL outcomes in LC patients were generally comparable to that of OC patients 5 years after diagnosis. Differences in HRQOL were observed only for body image, with LC patients reporting significantly better body image. No significant differences were noted for symptom burden. In terms of psychological well-being, no significant differences in distress were noted. LC patients were also less likely to be burdened by the treatment and the time after treatment completion.

Our results on HRQOL are generally in line with previous studies. Similar to a study on the short-term outcomes of LC patients [37], LC patients in our study were more satisfied with their body. With regards to comparison of long-term outcomes, our results are generally comparable to that reported in the LAFA trial [17]. In that study, no differences in HRQOL were found between LC patients and OC patients 2 to 5 years after surgery. Our study found differences only in body image. Possible explanation for this difference in results could be due to differences in samples. Our matched sample was younger than that in the LAFA trial. Evidence indicates that significant differences in HRQOL were more often found among younger colorectal cancer survivors [38]. Furthermore, we previously found that younger (< 50 years at diagnosis) colon cancer survivors reported significantly lower body image than older survivors 5–16 years after diagnosis [39].

Our results have clinical implications. LC patients were less burdened by the treatment and recovery time. These results support a previous study that reported patients treated for colorectal cancer consider being free of surgical complication and free of cancer as important factors in the post-operative period [20]. Although in the matched sample, disease recurrence was significantly higher in the LC group, nevertheless the proportion of LC patients reporting moderate/high fear of disease progression was not significantly higher. As LC is increasingly adopted as a feasible alternative to OC, it is important for patients to be aware that while LC can offer short- and long-term HRQOL benefits, there could be an increased risk of disease recurrence which can negatively impact HRQOL [40]. The increased risk of recurrence is disquieting and requires careful clarification in future research. This is relevant as the HRQOL in the LC group was at least comparable to OC, and LC is associated with comparable or better survival in clinical trials and population-based studies [3, 8].

To our knowledge, this is the first study to report on the long-term HRQOL of a population-based sample of LC and OC patients who completed a validated HRQOL assessment at uniform follow-up. Despite these strengths, there are several limitations which need to be discussed. Although the sample size as a whole was large, the proportion of LC patients was small (9%). This prevalence might be lower than other countries in Europe, but it is comparable to other reports using German samples [6, 8]. Furthermore, to reduce potential treatment selection bias, we used propensity score matching which reduced the OC sample significantly. However, comparable results from sensitivity analyses using the unmatched sample suggest that analyses from a smaller matched sample did not result in larger *p* values. Although we included a wide range of demographic and clinical variables into the propensity score, residual confounding could still exist as we do not have data on patients’ baseline performance status. Overall, we observed a tendency towards better HRQOL in LC patients for many subscales, which were, however, not statistically significant except for the body image subscale. Further larger studies, which have sufficient power to detect differences of the magnitude observed here, are needed to investigate whether our pattern only reflects a chance finding or a true difference between LC and OC patients.

In conclusion, among stage I-III colon patients, LC patients reported comparable long-term HRQOL outcomes and higher levels of psychological well-being than OC patients 5 years after diagnosis even though LC was associated with higher risk of disease recurrence.

## References

[1] Braga M (2005). Laparoscopic vs. open colectomy in cancer patients: long-term complications, quality of life, and survival. Dis Colon Rectum.

[2] Kuhry E (2008). Long‐term results of laparoscopic colorectal cancer resection. Cochrane Database Syst Rev.

[3] Ohtani H (2011). A meta-analysis of the short- and long-term results of randomized controlled trials that compared laparoscopy-assisted and conventional open surgery for colorectal cancer. J Cancer.

[4] Quintana JM (2018). Outcomes of open versus laparoscopic surgery in patients with colon cancer. Eur J Surg Oncol.

[5] Juo Y (2014). Is minimally invasive colon resection better than traditional approaches? First comprehensive national examination with propensity score matching. JAMA Surg.

[6] Benz S (2017). Laparoscopic surgery in patients with colon cancer: a population-based analysis. Surg Endosc.

[7] Stormark K (2016). Nationwide implementation of laparoscopic surgery for colon cancer: short-term outcomes and long-term survival in a population-based cohort. Surg Endosc.

[8] Babaei M, et al. Minimally invasive colorectal cancer surgery in Europe: implementation and outcomes. Medicine 2016;95:e3812-e. 10.1097/MD.0000000000003812 PMC4900730 27258522

[9] Janson M (2007). Randomized trial of health-related quality of life after open and laparoscopic surgery for colon cancer. Surg Endosc.

[10] Matsumoto S (2016). Prospective study of patient satisfaction and postoperative quality of life after laparoscopic colectomy in Japan. Asian J Endosc Surg.

[11] Stucky C-CH (2011). Long-term follow-up and individual item analysis of quality of life assessments related to laparoscopic-assisted colectomy in the COST trial 93-46-53 (INT 0146). Ann Surg Oncol.

[12] McCombie AM (2018). The ALCCaS trial: a randomized controlled trial comparing quality of life following laparoscopic versus open colectomy for colon cancer. Dis Colon Rectum.

[13] Theodoropoulos GE (2013). Prospective evaluation of health-related quality of life after laparoscopic colectomy for cancer. Tech Coloproctol.

[14] Michalopoulos NV (2013). A cost-utility analysis of laparoscopic vs open treatment of colorectal cancer in a public hospital of the Greek National Health System. J BUON.

[15] Vlug MS (2011). Laparoscopy in combination with fast track multimodal management is the best perioperative strategy in patients undergoing colonic surgery: a randomized clinical trial (LAFA-study). Ann Surg.

[16] Weeks JC (2002). Short-term quality-of-life outcomes following laparoscopic-assisted colectomy vs open colectomy for colon cancer: a randomized trial. JAMA.

[17] Bartels SAL (2014). Small bowel obstruction, incisional hernia and survival after laparoscopic and open colonic resection (LAFA study). Br J Surg.

[18] Mar J (2018). Cost-effectiveness analysis of laparoscopic versus open surgery in colon cancer. Surg Endosc.

[19] Liao C-H (2017). Real-world cost-effectiveness of laparoscopy versus open colectomy for colon cancer: a nationwide population-based study. Surg Endosc.

[20] Wrenn SM (2018). Patient perceptions and quality of life after colon and rectal surgery: What do patients really want?. Dis Colon Rectum.

[21] Franklin BR, McNally MP (2017). Laparoscopy for colon cancer. Clin Colon Rectal Surg.

[22] Fisher CS (2012). Fear of Recurrence and perceived survival benefit are primary motivators for choosing mastectomy over breast-conservation therapy regardless of age. Ann Surg Oncol.

[23] Brenner H (2011). Protection from colorectal cancer after colonoscopy: a population-based, case–control study. Ann Int Med.

[24] Aaronson NK (1993). The European Organization for Research and Treatment of Cancer QLQ-C30: a quality-of-life instrument for use in international clinical trials in oncology. J Natl Cancer Inst.

[25] Gujral S (2007). Assessing quality of life in patients with colorectal cancer: an update of the EORTC quality of life questionnaire. Eur J Cancer.

[26] Whistance RN (2009). Clinical and psychometric validation of the EORTC QLQ-CR29 questionnaire module to assess health-related quality of life in patients with colorectal cancer. Eur J Cancer.

[27] Fayers PM (1995). EORTC QLQ-C30 scoring manual.

[28] Book K (2011). Distress screening in oncology—evaluation of the Questionnaire on Distress in Cancer Patients—short form (QSC-R10) in a German sample. Psychooncology.

[29] Sturmer T (2014). Propensity scores for confounder adjustment when assessing the effects of medical interventions using nonexperimental study designs. J Intern Med.

[30] Ratnapradipa KL (2017). Patient, hospital, and geographic disparities in laparoscopic surgery use among surveillance, epidemiology, and end results-medicare patients with colon cancer. Dis Colon Rectum.

[31] Aarts MJ (2010). Socioeconomic status and changing inequalities in colorectal cancer? A review of the associations with risk, treatment and outcome. Eur J Cancer.

[32] Akinyemiju T (2016). Race/ethnicity and socio-economic differences in colorectal cancer surgery outcomes: analysis of the nationwide inpatient sample. BMC Cancer.

[33] Faries D (2010). Analysis of observational health care data using SAS.

[34] Austin PC (2010). Statistical criteria for selecting the optimal number of untreated subjects matched to each treated subject when using many-to-one matching on the propensity score. Am J Epidemiol.

[35] Stuart EA (2010). Matching methods for causal inference: a review and a look forward. Stat Sci.

[36] Burden A (2017). An evaluation of exact matching and propensity score methods as applied in a comparative effectiveness study of inhaled corticosteroids in asthma. Pragmat Obs Res.

[37] Scarpa M (2009). Minimally invasive surgery for colorectal cancer: quality of life, body image, cosmesis, and functional results. Surg Endosc.

[38] Bailey CE (2015). Functional deficits and symptoms of long-term survivors of colorectal cancer treated by multimodality therapy differ by age at diagnosis. J Gastrointest Surg.

[39] Thong MSY (2019). Age at diagnosis and gender are associated with long-term deficits in disease-specific health-related quality of life of colon and rectal cancer survivors: a population-based study. Dis Colon Rectum.

[40] Thong MS (2009). The impact of disease progression on perceived health status and quality of life of long-term cancer survivors. J Cancer Surviv.

