# Correction to: Association of laparoscopic colectomy versus open colectomy on the long‑term health‑related quality of life of colon cancer survivors

**DOI:** 10.1007/s00464-021-08557-0

**Published:** 2021-05-25

**Authors:** Melissa S. Y. Thong, Lina Jansen, Jenny Chang‑Claude, Michael Hofmeister, Hermann Brenner, Volker Arndt

**Affiliations:** 1 Unit of Cancer Survivorship, Division of Clinical, Epidemiology and Aging Research, German Cancer Research Center (DKFZ), P.O. Box 101949, 69009 Heidelberg, Germany; 2 Division of Clinical Epidemiology and Aging Research, German Cancer Research Center (DKFZ), Heidelberg, Germany; 3 Division of Cancer Epidemiology, German Cancer Research, Center (DKFZ), Heidelberg, Germany; 4 Genetic Tumour Epidemiology Group, University Medical Center Hamburg-Eppendorf, University Cancer Center Hamburg, Hamburg, Germany; 5 Division of Preventive Oncology, German Cancer Research Center (DKFZ) and National Center for Tumor Diseases (NCT), Heidelberg, Germany; 6 German Cancer Consortium (DKTK), German Cancer Research Center (DKFZ), Heidelberg, Germany

## Correction to: Surgical Endoscopy (2020) 34:5593–5603 10.1007/s00464-019-07360-2

The article “Association of laparoscopic colectomy versus open colectomy on the long‑term health‑related quality of life of colon cancer,” written by Melissa S. Y. Thong, Lina Jansen, Jenny Chang‑Claude, Michael Hofmeister, Hermann Brenner, and Volker Arndt, was originally published electronically on the publisher’s internet portal on 28 January 2020 without open access. With the author(s)’ decision to opt for Open Choice the copyright of the article changed on 29 April 2021 to © The Author(s) 2020 and the article is forthwith distributed under the terms of the Creative Commons Attribution 4.0 International License, which permits use, sharing, adaptation, distribution and reproduction in any medium or format, as long as you give appropriate credit to the original author(s) and the source, provide a link to the Creative Commons licence, and indicate if changes were made. The images or other third party material in this article are included in the article’s Creative Commons licence, unless indicated otherwise in a credit line to the material. If material is not included in the article’s Creative Commons licence and your intended use is not permitted by statutory regulation or exceeds the permitted use, you will need to obtain permission directly from the copyright holder. To view a copy of this licence, visit http://creativecommons.org/licenses/by/4.0.

